# Tetra-μ_3_-hydroxido-tetra­kis­[tricarbonyl­rhenium(I)] pyridine tetra­solvate

**DOI:** 10.1107/S1600536812036033

**Published:** 2012-08-25

**Authors:** M. Schutte, A. Brink, H. G. Visser, A. Roodt

**Affiliations:** a Department of Chemistry, University of the Free State, PO Box 339, Bloemfontein 9301, South Africa

## Abstract

The title compound, [Re_4_(μ_3_-OH)_4_(CO)_12_]·4C_5_H_5_N, crystallizes with one tetranuclear rhenium(I) cubane-like molecule and four pyridine mol­ecules in the asymmetric unit. The coordination environment of each Re^I^ atom is distorted octahedral. Four intra­molecular O—H⋯N and four inter­molecular C—H⋯O hydrogen-bond inter­actions are observed. Relatively strong hydrogen bonds are found between the hydrogen-bond donor (μ_3_-OH) and acceptor (basic N atom of pyridine), with N⋯O distances between 2.586 (10) and 2.628 (10) Å. Inter­cube distances of 9.873 (2) and 12.376 (3) Å are observed.

## Related literature

For similar structures, see: Herberhold & Süss (1975 ▶); Nuber *et al.* (1981 ▶); Copp *et al.* (1995 ▶); Egli *et al.* (1997 ▶). For the synthesis of the precursor, see: Alberto *et al.* (1996 ▶).
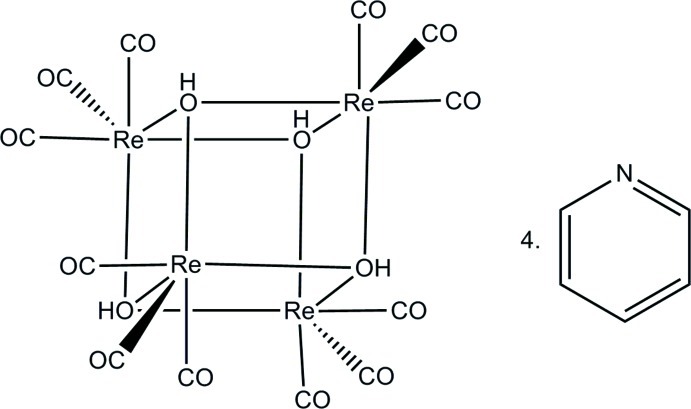


## Experimental

### 

#### Crystal data

[Re_4_(OH)_4_(CO)_12_]·4C_5_H_5_N
*M* *_r_* = 1465.39
Monoclinic, 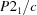

*a* = 11.895 (5) Å
*b* = 21.847 (5) Å
*c* = 16.245 (5) Å
β = 109.707 (5)°
*V* = 3974 (2) Å^3^
*Z* = 4
Mo *K*α radiation
μ = 12.22 mm^−1^
*T* = 100 K
0.43 × 0.11 × 0.11 mm

#### Data collection

Bruker APEXII CCD diffractometer
Absorption correction: multi-scan (*SADABS*; Bruker, 2008 ▶) *T*_min_ = 0.211, *T*_max_ = 0.271
68439 measured reflections
9594 independent reflections
7966 reflections with *I* > 2σ(*I*)
*R*_int_ = 0.064

#### Refinement

*R*[*F* ^2^ > 2σ(*F* ^2^)] = 0.043
*wR*(*F* ^2^) = 0.099
*S* = 1.04
9594 reflections
516 parameters
4 restraints
H atoms treated by a mixture of independent and constrained refinement
Δρ_max_ = 7.00 e Å^−3^
Δρ_min_ = −6.09 e Å^−3^

### 

Data collection: *APEX2* (Bruker, 2008 ▶); cell refinement: *SAINT-Plus* (Bruker, 2008 ▶); data reduction: *SAINT-Plus*; program(s) used to solve structure: *SHELXS97* (Sheldrick, 2008 ▶); program(s) used to refine structure: *SHELXL97* (Sheldrick, 2008 ▶); molecular graphics: *DIAMOND* (Brandenburg & Putz, 2005 ▶); software used to prepare material for publication: *WinGX* (Farrugia, 1999 ▶).

## Supplementary Material

Crystal structure: contains datablock(s) global, I. DOI: 10.1107/S1600536812036033/hg5243sup1.cif

Structure factors: contains datablock(s) I. DOI: 10.1107/S1600536812036033/hg5243Isup2.hkl

Additional supplementary materials:  crystallographic information; 3D view; checkCIF report

## Figures and Tables

**Table 1:** Selected bond lengths (Å)

Re3—C32	1.886 (10)
Re3—C31	1.900 (9)
Re3—C33	1.906 (10)
Re3—O1	2.157 (6)
Re3—O3	2.168 (6)
Re3—O4	2.179 (6)
Re4—C43	1.893 (9)
Re4—C42	1.913 (10)
Re4—C41	1.918 (9)
Re4—O2	2.154 (7)
Re4—O3	2.172 (6)
Re4—O4	2.173 (6)
Re2—C23	1.891 (9)
Re2—C22	1.916 (9)
Re2—C21	1.918 (10)
Re2—O3	2.157 (6)
Re2—O2	2.165 (6)
Re2—O1	2.171 (6)
O1—Re1	2.178 (6)
O4—Re1	2.160 (6)
Re1—C12	1.888 (10)
Re1—C13	1.889 (10)
Re1—C11	1.907 (10)
Re1—O2	2.169 (6)

**Table 2:** Hydrogen-bond geometry (Å, °)

*D*—H⋯*A*	*D*—H	H⋯*A*	*D*⋯*A*	*D*—H⋯*A*
C103—H103⋯O23^i^	0.93	2.59	3.249 (12)	128
C203—H203⋯O33^ii^	0.93	2.42	3.330 (13)	166
C303—H303⋯O42^iii^	0.93	2.49	3.321 (13)	149
C404—H404⋯O32^iv^	0.93	2.47	3.252 (12)	142
O1—H1⋯N2	0.85 (2)	1.76 (4)	2.600 (10)	170 (16)
O2—H2⋯N3	0.85 (2)	1.74 (3)	2.586 (10)	174 (11)
O3—H3⋯N1	0.85 (2)	1.79 (4)	2.627 (10)	165 (14)
O4—H4⋯N4	0.85 (2)	1.78 (4)	2.620 (10)	169 (15)

Symmetry codes: (i) 
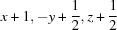
; (ii) 
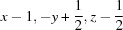
; (iii) 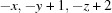; (iv) 
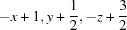
.

**Fig. 1.**
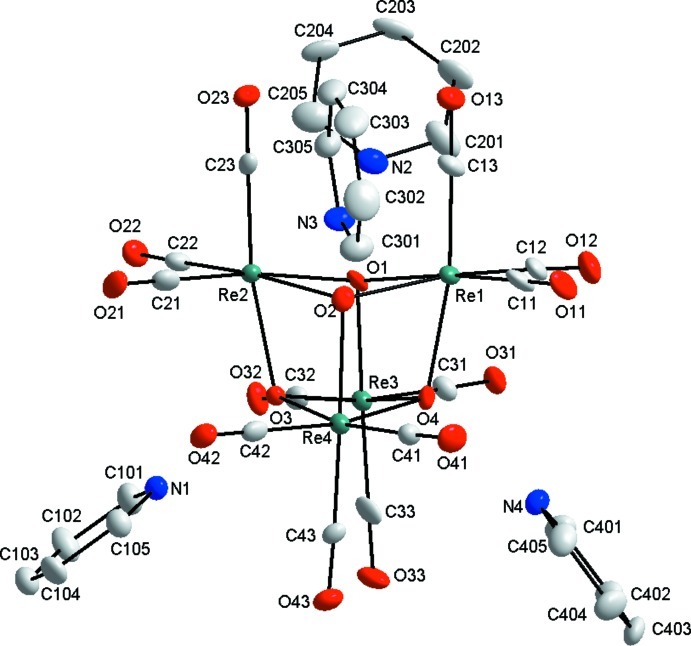
Representation of the title compound, showing the numbering scheme and displacement ellipsoids (50% probability).

**Fig. 2.**
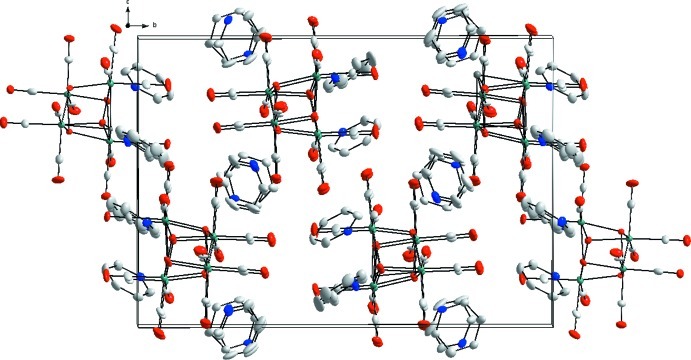
Packing of the title compound in the unit cell.

## supplementary crystallographic information

### Comment

The compound [Re_4_(CO)_12_(µ_3_-OH)_4_], which is a cubane-like molecule, crystallized with four pyridine ligands (hydrogen bond acceptor molecules or 'spacers') in the asymmetric unit. Four intramolecular hydrogen bonds are observed between the hydroxide bridges in the rhenium cluster and the pyridine molecules. These hydrogen interactions are also found by Copp *et al.* (1995) with benzene, toluene, *p*-xylene, *p*-fluorotoluene, naphthalene, 1-methylnaphthalene *etc*. surrounding the rhenium cluster as well as DMF and OPPh_3_ reported by Egli *et al.* (1997). Four intermolecular hydrogen interactions are reported between an aromatic carbon of the pyridine molecule to a carbonyl oxygen atom of the next rhenium cluster. The Re—O—Re angles (102.6 (3) ° to 104.7 (3) °) and the Re—O distances (2.154 (7) Å to 2.179 (6) Å) compare well to the structures of Herberhold & Süss (1975), Nuber *et al.* (1981), Copp *et al.* (1995) and Egli *et al.* (1997). The hydrogen bonds between the hydrogen-bond donor (µ_3_-OH) and acceptor (basic nitrogen of pyridine) are relatively strong with N···O distances ranging from 2.586 (10) Å to 2.628 (10) Å. This is comparable to the manganese structure by Copp *et al.* (1995) with a C···O distance of 2.74 Å from the bipyridine ligand to the bridging hydroxide oxygen. However, it does not correspond that well to the structure reported by Copp *et al.* (1995) with a distance of 3.58/3.59 Å for the C···O distance of the manganese structure with benzene as 'spacer'. Intercube distances of 9.873 (2) Å and 12.376 (3) Å are reported and fits well within the range found by Copp *et al.* (1995) (9.74 Å - 15.35 Å).

### Experimental

[NEt_4_]_2_[Re(CO)_3_Br_3_] (75 mg, 0.097 mmol), as prepared by Alberto *et al.* (1996), was dissolved in 20 ml of pyridine. The mixture was stirred at room temperature for 3 h and left to stand. The colourless cuboidal crystals formed by evaporation after a few days.

### Refinement

Aromatic and hydroxyl H atoms were positioned geometrically and allowed to ride on their parent atoms, with *U*_iso_(H) = 1.2*U*_eq_(parent) and *U*_iso_(H) = 1.5*U*_eq_(parent) of the parent atom with a C—H and O—H distance of 0.93 Å and 0.85 (2) Å respectively.

### Crystal data

**Table:**

[Re_4_(OH)_4_(CO)_12_]·4C_5_H_5_N	*F*(000) = 2688
*M**_r_* = 1465.39	*D*_x_ = 2.449 Mg m^−^^3^
Monoclinic, *P*2_1_/*c*	Mo *K*α radiation, λ = 0.71073 Å
Hall symbol: -P 2ybc	Cell parameters from 9793 reflections
*a* = 11.895 (5) Å	θ = 2.7–28.1°
*b* = 21.847 (5) Å	µ = 12.22 mm^−^^1^
*c* = 16.245 (5) Å	*T* = 100 K
β = 109.707 (5)°	Cuboid, colourless
*V* = 3974 (2) Å^3^	0.43 × 0.11 × 0.11 mm
*Z* = 4	

### Data collection

**Table:**

Bruker APEXII CCD diffractometer	7966 reflections with *I* > 2σ(*I*)
Graphite monochromator	*R*_int_ = 0.064
φ and ω scans	θ_max_ = 28°, θ_min_ = 2.6°
Absorption correction: multi-scan (*SADABS*; Bruker, 2008)	*h* = −15→15
*T*_min_ = 0.211, *T*_max_ = 0.271	*k* = −28→28
68439 measured reflections	*l* = −20→21
9594 independent reflections	

### Refinement

**Table:**

Refinement on *F*^2^	Primary atom site location: structure-invariant direct methods
Least-squares matrix: full	Secondary atom site location: difference Fourier map
*R*[*F*^2^ > 2σ(*F*^2^)] = 0.043	Hydrogen site location: inferred from neighbouring sites
*wR*(*F*^2^) = 0.099	H atoms treated by a mixture of independent and constrained refinement
*S* = 1.04	*w* = 1/[σ^2^(*F*_o_^2^) + (0.0181*P*)^2^ + 118.6736*P*] where *P* = (*F*_o_^2^ + 2*F*_c_^2^)/3
9594 reflections	(Δ/σ)_max_ = 0.001
516 parameters	Δρ_max_ = 7.00 e Å^−^^3^
4 restraints	Δρ_min_ = −6.09 e Å^−^^3^

### Special details

**Table:**

Geometry. All s.u.'s (except the s.u. in the dihedral angle between two l.s. planes) are estimated using the full covariance matrix. The cell s.u.'s are taken into account individually in the estimation of s.u.'s in distances, angles and torsion angles; correlations between s.u.'s in cell parameters are only used when they are defined by crystal symmetry. An approximate (isotropic) treatment of cell s.u.'s is used for estimating s.u.'s involving l.s. planes.
Refinement. Refinement of *F*^2^ against ALL reflections. The weighted *R*-factor *wR* and goodness of fit *S* are based on *F*^2^, conventional *R*-factors *R* are based on *F*, with *F* set to zero for negative *F*^2^. The threshold expression of *F*^2^ > 2σ(*F*^2^) is used only for calculating *R*-factors(gt) *etc*. and is not relevant to the choice of reflections for refinement. *R*-factors based on *F*^2^ are statistically about twice as large as those based on *F*, and *R*- factors based on ALL data will be even larger.

### Fractional atomic coordinates and isotropic or equivalent isotropic displacement parameters (Å^2^)

**Table:**

	*x*	*y*	*z*	*U*_iso_*/*U*_eq_	
Re3	0.36392 (3)	0.330517 (15)	0.69816 (2)	0.01378 (7)	
Re4	0.36579 (3)	0.431796 (15)	0.85997 (2)	0.01378 (7)	
Re2	0.16248 (3)	0.316708 (15)	0.80536 (2)	0.01277 (8)	
O1	0.1755 (6)	0.3368 (3)	0.6780 (4)	0.0146 (12)	
O33	0.6355 (6)	0.3461 (4)	0.7514 (4)	0.0299 (17)	
C23	−0.0056 (8)	0.3095 (4)	0.7695 (5)	0.0146 (17)	
O4	0.3333 (6)	0.4265 (3)	0.7203 (4)	0.0154 (12)	
O23	−0.1098 (6)	0.3044 (3)	0.7458 (4)	0.0235 (14)	
Re1	0.14174 (3)	0.434793 (15)	0.66166 (2)	0.01489 (8)	
O3	0.3485 (5)	0.3351 (3)	0.8273 (4)	0.0128 (12)	
O41	0.3519 (6)	0.5714 (3)	0.8711 (5)	0.0258 (15)	
O42	0.3772 (6)	0.4234 (3)	1.0504 (4)	0.0217 (14)	
O43	0.6378 (6)	0.4340 (3)	0.9300 (4)	0.0257 (15)	
C43	0.5348 (8)	0.4345 (4)	0.9030 (5)	0.0147 (17)	
C42	0.3727 (8)	0.4268 (4)	0.9792 (6)	0.0195 (19)	
C41	0.3593 (8)	0.5192 (4)	0.8685 (6)	0.0178 (18)	
O31	0.3407 (7)	0.3349 (3)	0.5051 (4)	0.0277 (16)	
O21	0.1723 (7)	0.3090 (4)	0.9962 (4)	0.0331 (18)	
C31	0.3503 (8)	0.3337 (4)	0.5783 (6)	0.0189 (18)	
C21	0.1686 (9)	0.3116 (4)	0.9247 (6)	0.022 (2)	
C33	0.5327 (9)	0.3392 (5)	0.7306 (5)	0.021 (2)	
O22	0.1678 (7)	0.1772 (3)	0.7977 (5)	0.0332 (17)	
C22	0.1671 (8)	0.2291 (4)	0.8013 (6)	0.0192 (18)	
O32	0.3974 (7)	0.1929 (3)	0.6811 (4)	0.0304 (17)	
O11	0.1210 (8)	0.5739 (3)	0.6689 (6)	0.045 (2)	
C32	0.3824 (9)	0.2454 (5)	0.6885 (6)	0.021 (2)	
C11	0.1278 (9)	0.5215 (5)	0.6665 (7)	0.027 (2)	
O12	0.1361 (7)	0.4482 (4)	0.4735 (5)	0.0336 (18)	
C12	0.1361 (9)	0.4431 (4)	0.5446 (6)	0.023 (2)	
C13	−0.0265 (9)	0.4291 (4)	0.6207 (6)	0.0206 (19)	
O13	−0.1292 (7)	0.4231 (3)	0.5947 (5)	0.0320 (17)	
N1	0.5054 (7)	0.2663 (4)	0.9429 (5)	0.0211 (17)	
N2	0.0085 (8)	0.2811 (4)	0.5546 (5)	0.0247 (18)	
N3	0.0328 (7)	0.4768 (4)	0.8558 (5)	0.0226 (17)	
N4	0.4824 (7)	0.4935 (4)	0.6715 (5)	0.0224 (17)	
C305	−0.0787 (10)	0.4596 (5)	0.8305 (7)	0.032 (2)	
H305	−0.0992	0.4236	0.7984	0.038*	
C302	−0.0225 (13)	0.5632 (6)	0.9231 (9)	0.047 (3)	
H302	0.0003	0.5985	0.9566	0.056*	
C304	−0.1692 (11)	0.4915 (6)	0.8484 (8)	0.040 (3)	
H304	−0.2472	0.4769	0.83	0.047*	
C303	−0.1390 (11)	0.5454 (6)	0.8941 (9)	0.042 (3)	
H303	−0.1971	0.5693	0.905	0.05*	
C301	0.0622 (11)	0.5286 (5)	0.9025 (8)	0.035 (3)	
H301	0.1412	0.5417	0.9216	0.042*	
C403	0.6711 (9)	0.5524 (5)	0.6397 (7)	0.026 (2)	
H403	0.7338	0.572	0.6285	0.031*	
C402	0.6276 (9)	0.4985 (5)	0.5998 (6)	0.024 (2)	
H402	0.6607	0.4807	0.5613	0.029*	
C401	0.5332 (9)	0.4701 (5)	0.6170 (6)	0.027 (2)	
H401	0.5043	0.4332	0.5893	0.032*	
C404	0.6207 (10)	0.5777 (5)	0.6972 (7)	0.029 (2)	
H404	0.6495	0.6142	0.7261	0.035*	
C405	0.5257 (10)	0.5471 (5)	0.7108 (7)	0.028 (2)	
H405	0.4905	0.5644	0.7485	0.034*	
C205	−0.0490 (10)	0.2326 (5)	0.5699 (7)	0.033 (2)	
H205	−0.0253	0.2164	0.6261	0.039*	
C201	−0.0277 (11)	0.3038 (5)	0.4733 (7)	0.035 (3)	
H201	0.0107	0.3382	0.462	0.042*	
C204	−0.1424 (11)	0.2052 (6)	0.5054 (9)	0.048 (4)	
H204	−0.181	0.1715	0.5184	0.058*	
C203	−0.1781 (12)	0.2284 (7)	0.4218 (9)	0.052 (4)	
H203	−0.2405	0.2105	0.3772	0.062*	
C202	−0.1194 (13)	0.2784 (7)	0.4058 (8)	0.053 (4)	
H202	−0.1412	0.295	0.35	0.063*	
C105	0.5525 (9)	0.2822 (5)	1.0269 (6)	0.023 (2)	
H105	0.5308	0.3196	1.0443	0.027*	
C103	0.6650 (9)	0.1902 (5)	1.0643 (6)	0.024 (2)	
H103	0.7184	0.1646	1.1048	0.029*	
C104	0.6325 (9)	0.2455 (5)	1.0898 (6)	0.027 (2)	
H104	0.6633	0.2581	1.1477	0.032*	
C102	0.6167 (10)	0.1732 (5)	0.9773 (6)	0.028 (2)	
H102	0.6372	0.136	0.9583	0.034*	
C101	0.5376 (10)	0.2124 (5)	0.9189 (6)	0.027 (2)	
H101	0.5055	0.2007	0.8606	0.032*	
O2	0.1773 (6)	0.4152 (3)	0.7990 (4)	0.0166 (13)	
H3	0.395 (10)	0.316 (6)	0.871 (6)	0.06 (4)*	
H2	0.134 (8)	0.436 (4)	0.821 (6)	0.02 (3)*	
H1	0.127 (11)	0.319 (6)	0.634 (6)	0.07 (5)*	
H4	0.376 (11)	0.452 (5)	0.704 (9)	0.07 (5)*	

### Atomic displacement parameters (Å^2^)

**Table:**

	*U*^11^	*U*^22^	*U*^33^	*U*^12^	*U*^13^	*U*^23^
Re3	0.01351 (13)	0.01541 (12)	0.01219 (11)	0.00124 (9)	0.00403 (9)	0.00039 (8)
Re4	0.01351 (13)	0.01541 (12)	0.01219 (11)	0.00124 (9)	0.00403 (9)	0.00039 (8)
Re2	0.01148 (17)	0.01394 (16)	0.01229 (15)	−0.00146 (12)	0.00322 (12)	−0.00020 (12)
O1	0.014 (3)	0.014 (3)	0.010 (3)	0.002 (2)	−0.003 (2)	0.000 (2)
O33	0.013 (4)	0.053 (5)	0.023 (3)	0.006 (3)	0.004 (3)	0.005 (3)
C23	0.017 (5)	0.015 (4)	0.010 (4)	−0.005 (3)	0.003 (3)	0.000 (3)
O4	0.019 (3)	0.014 (3)	0.016 (3)	0.001 (3)	0.009 (3)	0.005 (2)
O23	0.016 (4)	0.022 (3)	0.032 (4)	−0.002 (3)	0.008 (3)	0.004 (3)
Re1	0.01131 (17)	0.01397 (16)	0.01761 (16)	0.00153 (13)	0.00254 (13)	0.00234 (12)
O3	0.013 (3)	0.013 (3)	0.011 (3)	−0.001 (2)	0.003 (2)	0.000 (2)
O41	0.028 (4)	0.012 (3)	0.034 (4)	−0.006 (3)	0.006 (3)	−0.004 (3)
O42	0.021 (4)	0.029 (4)	0.017 (3)	−0.006 (3)	0.009 (3)	−0.003 (3)
O43	0.017 (4)	0.030 (4)	0.030 (4)	−0.007 (3)	0.008 (3)	−0.001 (3)
C43	0.015 (4)	0.014 (4)	0.016 (4)	−0.004 (3)	0.007 (3)	−0.004 (3)
C42	0.015 (5)	0.013 (4)	0.029 (5)	−0.003 (3)	0.006 (4)	−0.001 (4)
C41	0.015 (4)	0.016 (4)	0.020 (4)	−0.001 (3)	0.003 (4)	0.002 (3)
O31	0.034 (4)	0.034 (4)	0.016 (3)	0.005 (3)	0.009 (3)	0.002 (3)
O21	0.038 (5)	0.049 (5)	0.016 (3)	−0.012 (4)	0.014 (3)	−0.002 (3)
C31	0.019 (5)	0.022 (4)	0.015 (4)	0.004 (4)	0.003 (3)	0.001 (3)
C21	0.016 (5)	0.019 (4)	0.027 (5)	−0.005 (4)	0.002 (4)	−0.002 (4)
C33	0.023 (5)	0.031 (5)	0.006 (4)	0.008 (4)	0.002 (3)	−0.001 (3)
O22	0.035 (4)	0.018 (4)	0.040 (4)	0.001 (3)	0.005 (3)	0.000 (3)
C22	0.012 (4)	0.018 (4)	0.023 (4)	0.000 (3)	0.001 (4)	0.000 (4)
O32	0.050 (5)	0.017 (3)	0.024 (3)	0.008 (3)	0.012 (3)	0.000 (3)
O11	0.047 (5)	0.013 (4)	0.056 (5)	0.003 (3)	−0.007 (4)	0.003 (3)
C32	0.019 (5)	0.029 (5)	0.014 (4)	−0.004 (4)	0.003 (4)	0.002 (4)
C11	0.016 (5)	0.025 (5)	0.027 (5)	0.003 (4)	−0.008 (4)	0.005 (4)
O12	0.033 (4)	0.039 (4)	0.026 (4)	0.006 (3)	0.006 (3)	0.016 (3)
C12	0.016 (5)	0.023 (5)	0.023 (5)	0.004 (4)	−0.001 (4)	0.010 (4)
C13	0.015 (5)	0.020 (4)	0.021 (4)	0.004 (4)	−0.001 (4)	0.000 (4)
O13	0.022 (4)	0.029 (4)	0.042 (4)	0.002 (3)	0.006 (3)	0.005 (3)
N1	0.020 (4)	0.027 (4)	0.015 (3)	−0.004 (3)	0.004 (3)	0.003 (3)
N2	0.022 (4)	0.022 (4)	0.026 (4)	0.000 (3)	0.003 (3)	−0.007 (3)
N3	0.018 (4)	0.022 (4)	0.029 (4)	0.003 (3)	0.009 (3)	−0.005 (3)
N4	0.022 (4)	0.024 (4)	0.023 (4)	−0.003 (3)	0.009 (3)	0.003 (3)
C305	0.029 (6)	0.032 (6)	0.038 (6)	0.001 (5)	0.016 (5)	0.004 (5)
C302	0.067 (10)	0.029 (6)	0.052 (7)	0.015 (6)	0.030 (7)	−0.011 (5)
C304	0.025 (6)	0.047 (7)	0.052 (7)	0.006 (5)	0.020 (5)	0.014 (6)
C303	0.037 (7)	0.041 (7)	0.057 (8)	0.015 (6)	0.029 (6)	0.000 (6)
C301	0.031 (6)	0.030 (6)	0.047 (7)	0.002 (5)	0.016 (5)	−0.007 (5)
C403	0.026 (5)	0.025 (5)	0.031 (5)	0.000 (4)	0.016 (4)	0.011 (4)
C402	0.019 (5)	0.034 (5)	0.022 (5)	−0.001 (4)	0.010 (4)	0.002 (4)
C401	0.026 (6)	0.031 (5)	0.022 (5)	−0.008 (4)	0.006 (4)	−0.006 (4)
C404	0.031 (6)	0.017 (5)	0.042 (6)	−0.005 (4)	0.015 (5)	−0.005 (4)
C405	0.034 (6)	0.025 (5)	0.034 (5)	0.007 (4)	0.021 (5)	0.002 (4)
C205	0.030 (6)	0.035 (6)	0.034 (6)	0.002 (5)	0.011 (5)	−0.015 (5)
C201	0.041 (7)	0.031 (6)	0.023 (5)	0.013 (5)	−0.001 (5)	−0.005 (4)
C204	0.033 (7)	0.045 (7)	0.069 (9)	−0.017 (6)	0.020 (7)	−0.035 (7)
C203	0.030 (7)	0.062 (9)	0.046 (7)	0.010 (6)	−0.009 (6)	−0.039 (7)
C202	0.054 (9)	0.067 (9)	0.024 (6)	0.016 (7)	−0.005 (6)	−0.024 (6)
C105	0.025 (5)	0.023 (5)	0.020 (4)	0.003 (4)	0.007 (4)	0.001 (4)
C103	0.025 (5)	0.024 (5)	0.022 (5)	0.004 (4)	0.006 (4)	0.005 (4)
C104	0.026 (6)	0.033 (5)	0.015 (4)	−0.001 (4)	−0.002 (4)	0.003 (4)
C102	0.035 (6)	0.029 (5)	0.022 (5)	0.015 (5)	0.010 (4)	0.003 (4)
C101	0.032 (6)	0.026 (5)	0.020 (5)	0.001 (4)	0.005 (4)	0.003 (4)
O2	0.021 (3)	0.015 (3)	0.017 (3)	0.001 (3)	0.010 (3)	−0.002 (2)

### Geometric parameters (Å, º)

**Table:**

Re3—C32	1.886 (10)	N2—C201	1.339 (13)
Re3—C31	1.900 (9)	N3—C305	1.304 (14)
Re3—C33	1.906 (10)	N3—C301	1.342 (13)
Re3—O1	2.157 (6)	N4—C401	1.331 (13)
Re3—O3	2.168 (6)	N4—C405	1.349 (13)
Re3—O4	2.179 (6)	C305—C304	1.392 (16)
Re4—C43	1.893 (9)	C305—H305	0.93
Re4—C42	1.913 (10)	C302—C303	1.362 (19)
Re4—C41	1.918 (9)	C302—C301	1.386 (16)
Re4—O2	2.154 (7)	C302—H302	0.93
Re4—O3	2.172 (6)	C304—C303	1.373 (18)
Re4—O4	2.173 (6)	C304—H304	0.93
Re2—C23	1.891 (9)	C303—H303	0.93
Re2—C22	1.916 (9)	C301—H301	0.93
Re2—C21	1.918 (10)	C403—C402	1.359 (14)
Re2—O3	2.157 (6)	C403—C404	1.385 (14)
Re2—O2	2.165 (6)	C403—H403	0.93
Re2—O1	2.171 (6)	C402—C401	1.392 (14)
O1—Re1	2.178 (6)	C402—H402	0.93
O1—H1	0.85 (2)	C401—H401	0.93
O33—C33	1.164 (12)	C404—C405	1.392 (15)
C23—O23	1.173 (11)	C404—H404	0.93
O4—Re1	2.160 (6)	C405—H405	0.93
O4—H4	0.85 (2)	C205—C204	1.380 (16)
Re1—C12	1.888 (10)	C205—H205	0.93
Re1—C13	1.889 (10)	C201—C202	1.376 (16)
Re1—C11	1.907 (10)	C201—H201	0.93
Re1—O2	2.169 (6)	C204—C203	1.38 (2)
O3—H3	0.85 (2)	C204—H204	0.93
O41—C41	1.146 (11)	C203—C202	1.37 (2)
O42—C42	1.143 (11)	C203—H203	0.93
O43—C43	1.154 (11)	C202—H202	0.93
O31—C31	1.156 (11)	C105—C104	1.391 (13)
O21—C21	1.149 (11)	C105—H105	0.93
O22—C22	1.137 (11)	C103—C104	1.375 (14)
O32—C32	1.172 (12)	C103—C102	1.386 (13)
O11—C11	1.149 (12)	C103—H103	0.93
O12—C12	1.161 (12)	C104—H104	0.93
C13—O13	1.157 (12)	C102—C101	1.384 (14)
N1—C105	1.334 (12)	C102—H102	0.93
N1—C101	1.338 (13)	C101—H101	0.93
N2—C205	1.328 (14)	O2—H2	0.85 (2)
			
C32—Re3—C31	85.6 (4)	O42—C42—Re4	179.5 (9)
C32—Re3—C33	88.8 (4)	O41—C41—Re4	177.6 (8)
C31—Re3—C33	89.8 (4)	O31—C31—Re3	178.9 (9)
C32—Re3—O1	101.2 (3)	O21—C21—Re2	179.5 (9)
C31—Re3—O1	96.7 (3)	O33—C33—Re3	178.0 (9)
C33—Re3—O1	168.4 (3)	O22—C22—Re2	178.7 (9)
C32—Re3—O3	100.1 (3)	O32—C32—Re3	177.3 (9)
C31—Re3—O3	169.7 (3)	O11—C11—Re1	178.9 (11)
C33—Re3—O3	98.8 (3)	O12—C12—Re1	178.1 (9)
O1—Re3—O3	73.8 (2)	O13—C13—Re1	177.2 (8)
C32—Re3—O4	173.6 (3)	C105—N1—C101	117.7 (8)
C31—Re3—O4	99.9 (3)	C205—N2—C201	117.8 (9)
C33—Re3—O4	94.5 (3)	C305—N3—C301	117.7 (9)
O1—Re3—O4	75.0 (2)	C401—N4—C405	117.5 (9)
O3—Re3—O4	73.9 (2)	N3—C305—C304	124.5 (11)
C43—Re4—C42	87.2 (4)	N3—C305—H305	117.8
C43—Re4—C41	90.3 (4)	C304—C305—H305	117.8
C42—Re4—C41	88.4 (4)	C303—C302—C301	119.8 (12)
C43—Re4—O2	170.3 (3)	C303—C302—H302	120.1
C42—Re4—O2	98.0 (3)	C301—C302—H302	120.1
C41—Re4—O2	98.0 (3)	C303—C304—C305	117.5 (11)
C43—Re4—O3	97.0 (3)	C303—C304—H304	121.3
C42—Re4—O3	99.2 (3)	C305—C304—H304	121.3
C41—Re4—O3	169.6 (3)	C302—C303—C304	118.9 (11)
O2—Re4—O3	74.1 (2)	C302—C303—H303	120.6
C43—Re4—O4	100.4 (3)	C304—C303—H303	120.6
C42—Re4—O4	170.3 (3)	N3—C301—C302	121.6 (11)
C41—Re4—O4	97.5 (3)	N3—C301—H301	119.2
O2—Re4—O4	73.7 (2)	C302—C301—H301	119.2
O3—Re4—O4	74.0 (2)	C402—C403—C404	119.0 (9)
C23—Re2—C22	86.9 (4)	C402—C403—H403	120.5
C23—Re2—C21	89.0 (4)	C404—C403—H403	120.5
C22—Re2—C21	89.2 (4)	C403—C402—C401	119.7 (9)
C23—Re2—O3	170.2 (3)	C403—C402—H402	120.2
C22—Re2—O3	98.7 (3)	C401—C402—H402	120.2
C21—Re2—O3	99.0 (3)	N4—C401—C402	122.7 (9)
C23—Re2—O2	99.4 (3)	N4—C401—H401	118.7
C22—Re2—O2	170.9 (3)	C402—C401—H401	118.7
C21—Re2—O2	97.5 (3)	C403—C404—C405	118.3 (9)
O3—Re2—O2	74.2 (2)	C403—C404—H404	120.8
C23—Re2—O1	97.5 (3)	C405—C404—H404	120.8
C22—Re2—O1	99.0 (3)	N4—C405—C404	122.8 (9)
C21—Re2—O1	169.8 (3)	N4—C405—H405	118.6
O3—Re2—O1	73.8 (2)	C404—C405—H405	118.6
O2—Re2—O1	73.8 (2)	N2—C205—C204	122.5 (12)
Re3—O1—Re2	104.3 (2)	N2—C205—H205	118.8
Re3—O1—Re1	102.7 (3)	C204—C205—H205	118.8
Re2—O1—Re1	104.2 (2)	N2—C201—C202	122.7 (13)
Re3—O1—H1	118 (10)	N2—C201—H201	118.6
Re2—O1—H1	117 (10)	C202—C201—H201	118.6
Re1—O1—H1	108 (10)	C203—C204—C205	119.4 (13)
O23—C23—Re2	178.7 (8)	C203—C204—H204	120.3
Re1—O4—Re4	104.2 (3)	C205—C204—H204	120.3
Re1—O4—Re3	102.6 (3)	C202—C203—C204	118.3 (11)
Re4—O4—Re3	104.0 (2)	C202—C203—H203	120.9
Re1—O4—H4	117 (10)	C204—C203—H203	120.9
Re4—O4—H4	112 (10)	C203—C202—C201	119.3 (13)
Re3—O4—H4	116 (10)	C203—C202—H202	120.3
C12—Re1—C13	88.7 (4)	C201—C202—H202	120.3
C12—Re1—C11	88.4 (4)	N1—C105—C104	123.2 (9)
C13—Re1—C11	89.0 (4)	N1—C105—H105	118.4
C12—Re1—O4	97.2 (3)	C104—C105—H105	118.4
C13—Re1—O4	170.0 (3)	C104—C103—C102	118.7 (9)
C11—Re1—O4	99.1 (3)	C104—C103—H103	120.7
C12—Re1—O2	169.5 (3)	C102—C103—H103	120.7
C13—Re1—O2	99.5 (3)	C103—C104—C105	118.6 (9)
C11—Re1—O2	98.2 (4)	C103—C104—H104	120.7
O4—Re1—O2	73.7 (2)	C105—C104—H104	120.7
C12—Re1—O1	99.2 (3)	C101—C102—C103	119.1 (9)
C13—Re1—O1	96.2 (3)	C101—C102—H102	120.5
C11—Re1—O1	170.8 (3)	C103—C102—H102	120.5
O4—Re1—O1	75.0 (2)	N1—C101—C102	122.8 (9)
O2—Re1—O1	73.5 (2)	N1—C101—H101	118.6
Re2—O3—Re3	104.4 (2)	C102—C101—H101	118.6
Re2—O3—Re4	103.2 (2)	Re4—O2—Re2	103.5 (3)
Re3—O3—Re4	104.4 (2)	Re4—O2—Re1	104.6 (3)
Re2—O3—H3	112 (10)	Re2—O2—Re1	104.7 (2)
Re3—O3—H3	123 (10)	Re4—O2—H2	114 (8)
Re4—O3—H3	107 (10)	Re2—O2—H2	115 (8)
O43—C43—Re4	177.6 (8)	Re1—O2—H2	113 (7)

### Hydrogen-bond geometry (Å, º)

**Table:**

*D*—H···*A*	*D*—H	H···*A*	*D*···*A*	*D*—H···*A*
C103—H103···O23^i^	0.93	2.59	3.249 (12)	128
C203—H203···O33^ii^	0.93	2.42	3.330 (13)	166
C303—H303···O42^iii^	0.93	2.49	3.321 (13)	149
C404—H404···O32^iv^	0.93	2.47	3.252 (12)	142
O1—H1···N2	0.85 (2)	1.76 (4)	2.600 (10)	170 (16)
O2—H2···N3	0.85 (2)	1.74 (3)	2.586 (10)	174 (11)
O3—H3···N1	0.85 (2)	1.79 (4)	2.627 (10)	165 (14)
O4—H4···N4	0.85 (2)	1.78 (4)	2.620 (10)	169 (15)

Symmetry codes: (i) *x*+1, −*y*+1/2, *z*+1/2; (ii) *x*−1, −*y*+1/2, *z*−1/2; (iii) −*x*, −*y*+1, −*z*+2; (iv) −*x*+1, *y*+1/2, −*z*+3/2.
